# Supplementary material for: Are carbon emissions trading and green financial instruments synergistic? -Comprehensive quantitative research based on content analysis
Source: PLoS One. 2024 Mar 7;19(3):e0298601. doi: 10.1371/journal.pone.0298601 (PMC10919601; doi:10.1371/journal.pone.0298601)
Supplement: S1 Data — (ZIP) [file pone.0298601.s001.zip › Data/地级市Z_F工作报告.zip!/░▓╗╒/╨√│╟╩╨/└┤╫╘╣1⁄2╓┌║┼í╛┬φ┐╦╩2╛▌═°í┐_2017.docx]

各位代表：

现在，我代表市人民政府，向大会报告政府工作，请予审议，并请市政协委员和列席会议的同志提出意见。

一、2016年和本届政府工作回顾

刚刚过去的2016年，面对复杂的宏观环境和罕见的洪涝灾害，全市上下在市委的坚强领导下，认真落实省委、省政府决策部署，顶住经济下行压力，保持发展定力，扎实做好各项工作，顺利完成市三届人大七次会议确定的目标任务，实现了“十三五”发展良好开局。初步测算，完成地区生产总值1057.8亿元，同比增长8.7%；财政收入202.4亿元，增长7.1%；规模以上工业增加值430.7亿元，增长9.4%；固定资产投资1414.3亿元，增长10.2%；社会消费品零售总额475.8亿元，增长12.6%；进出口总额15.1亿美元；城镇居民人均可支配收入30877元，增长8%；农村居民人均可支配收入13379元，增长8.7%。

一年来，我们着重做了以下工作：

（一）扎实推进供给侧结构性改革，实体经济进一步发展。积极主动减量。省定33万吨过剩产能化解任务提前完成，职工得到妥善安置。分类处置“僵尸企业”40户。商品住宅去化周期比全省短3.4个月。着力降本增效。全面落实减税降费政策，累计减少企业支出4.8亿元，减免退税43.7亿元。规模以上工业企业利润增长9%。畅通金融血脉。在全省首创出口退税贷，税融通和“4321”政银担业务量分别居全省第2位、第4位，新增贷款144.7亿元。黄山胶囊A股首发上市，华菱精工顺利报会，9家企业“新三板”挂牌，直接融资81.9亿元。扩大有效投资。287个列入省亿元以上重点项目完成投资559.7亿元，项目开工率、竣工率、投资完成率均居全省前列。±1100千伏古泉换流站、绩溪抽水蓄能电站、亚太汽车关键零部件、鼎连混炼胶等项目加快建设，中鼎动力、卓越新能源等项目竣工投产。

（二）加快转型升级步伐，产业结构进一步优化。新型工业化加速推进。启动实施中国制造2025宣城行动。汽车和装备制造、食品医药、新材料三大工业主导产业产值占比提高4.6个百分点。宁国核心基础零部件基地在省预警评比中全部绿灯达标，首批5个市级基地建设扎实推进。实现战略性新兴产业产值485.1亿元，增长11.5%；高新技术产业增加值187.6亿元，增长22.5%。完成工业技改投资367.9亿元，增长49.5%，居全省第1位。新增国家级两化融合管理体系贯标试点企业2户、省级10户，分别居全省第2位和第5位。中鼎集团入围工信部制造业单项冠军示范企业。江南化工成为国家智能制造试点示范项目企业。质量工作得到国务院考核组充分肯定。2个项目荣获“质量之光”奖，2人跻身“大国工匠”行列；司尔特肥业公司荣获省政府质量奖。新增省级标准化示范企业数居全省第1位；新认定安徽工业精品6件，新注册商标1414件。现代服务业发展提速。服务业增加值427.9亿元，增长11.1%。新增省级现代服务业集聚区2个。游客接待量突破2550万人次，增长18.2%，旅游总收入突破200亿元，增长18.6%，增幅分别居全省第3位和第1位。宣城文化旅游发展股份有限公司挂牌成立。成为国家全域旅游示范区创建单位和中国最佳文化旅游目的地、健康养生休闲度假旅游最佳目的地。旌德旌阳镇成为安徽首个、全国第六个“国际慢城”。宣纸文化园跻身全国首批研学旅游示范基地。徽杭古道居中国体育旅游十佳精品线路首位。“安徽川藏线”成为自驾游新热线。现代农业发展保持全省领先。实现农产品加工产值735亿元。新增农民专业合作社310家、家庭农场1160个。新增农业产业化国家重点龙头企业2家，7家企业进入省龙头企业“甲级队”。创建全国四星级休闲农业示范点8个。宣州、郎溪分别成为全国首批农村产业融合发展试点示范县和“新农直报平台”试点县。

（三）*推进改革创新，发展动力进一步增强。科创能力不断提升。科技创新潜力跃居全省第4位。新增国家技术创新示范企业1户、省级3户，新增省级企业技术中心6家、博士后科研工作站3家，规模以上工业企业研发机构覆盖率提高4.3个百分点，居全省第5位。宁国龙晟科技公司纤维状储能器件项目列入国家重点研发计划。成立宣城先进制造业技术服务中心。授权发明专利增长88.9%。引进落户高层次科创团队25个，其中获省扶持团队7个，并列全省第一；招引“国家千人计划”人才8人。“四新经济”加速成长。在全省率先启动特色小镇培育创建，旌德白地镇入选首批中国特色小镇，绩溪家朋乡获全国首家“摄影小镇”称号。电子商务加快发展，首次跻身全国电商百佳城市。阿里巴巴宣城·黄山区域跨境电商服务中心和区域性电商平台“上街去”正式运行，泾县李园村成为全省首个“淘宝村”。宣城智谷软件产业园正式开园。建成“0563创客街”等众创空间9家；入选首批国家级“星创天地”6家，居全省前列。创成全省首个国家级海峡两岸青年就业创业示范点。改革红利逐步**。深化“放管服”改革，积极推行“五证合一”和“两证整合”改革，新登记市场主体29069个，增长16.5%。“营改增”试点顺利完成。不动产登记制度改革工作走在全省前列。完成市县公共资源交易平台整合，建成公共资源交易、服务、监管电子化系统。农村土地承包经营权确权登记颁证工作全面完成，旌德县省级农村“三变”改革试点工作受到中央领导肯定。国有林场改革稳步实施。宣州区农民住房财产权抵押贷款试点扎实推进。大力促进民间投资，落地PPP项目17个，入围国家第三批PPP示范项目2个。广德撤县设市工作稳步推进。开放合作取得进展。学习对接沪苏浙，与上海松江区、闵行区和湖州市签订深化合作协议。郎溪、广德与溧阳签订共建苏皖合作示范区框架协议。强化招商引资，新签亿元以上项目270个，到位省外资金770.2亿元，居全省第3位；实际利用外资8亿美元，增长14%，增幅居全省第4位；新建项目主导产业契合度达65%。在中国国际徽商大会上成功签约项目34个，投资额327.5亿元。建成外贸综合服务平台和全省首个公路无水港。中鼎密封件获“中国出口质量安全示范企业”称号，5家食品出口企业成为国家认监委“同线同标同质”推荐企业。大力支持企业“走出去”，中鼎集团入选海外并购金哨奖“中国十佳大买手”。

（四）加强基础设施建设，城乡面貌进一步改观。中心城市发展加快。正式成为长三角城市群和“芜马宣”城市组群成员。宣城经开区发展提速，新开工工业项目32个、投产20个，以锂电池为主导的新能源新材料产业集群效应初显，利用世行贷款项目稳步推进。敬亭山旅游度假区5A景区创建工作扎实开展。宣城现代服务业产业园区交易额达百亿，新塘羽绒产业园建设加快，建材（钢材）物流交易中心、电子商务与快递业协同发展集中区等项目开工建设。《宣城市环南漪湖生态保护区概念性总体规划》在联合国人居大会上获高度评价。成为城市总体规划修编省级创新试点和全省城市设计工作试点市。城市建设续建新建项目210个，完成投资132亿元，其中市政公用设施建设投资66.4亿元。市植物园、九曲公园、敬亭圩污水处理厂二期等项目建成，水阳江大道闭合段、市人民医院改扩建等项目提速，市全民健身中心、游泳馆、宛陵湖东湖公园、万达广场和首批地下综合管廊项目开工建设。市区完成征地5060亩，房屋征收2985户、48万平方米，拆除违法建设10万平方米。智慧城市信息化基础设施建设全省领先，宣城政务云计算中心投入使用。交通建设会战成效显著。溧广高速安徽段正式通车，扬绩高速鸿门互通顺利建成，宣狸高速建设加快，广宁高速开工建设。建成省道S104平兴段一级公路，完成国省道路面改善100公里，干线公路好路率保持全省第一；建成农村道路畅通工程2500公里。水阳江航道整治和宣州综合码头工程加快推进。商合杭铁路宣城段、皖赣铁路扩能改造芜宣段、杭黄铁路绩溪段、芜湖至广德铁路电气化改造等项目进展顺利。芜宣机场正式获批并开工建设试验段。水利电力等建设得到加强。全面完成水利水毁修复，大力推进防洪薄弱环节治理，水利建设投资完成率居全省首位。青弋江综合治理、水阳江生态蓄水和城东联圩、双桥联圩防洪治理等工程及扬溪源水库开工建设。续建新建110千伏及以上电网工程17个、竣工投产9个，农电配网改造升级惠及4.3万低压用户。百兆光纤实现自然村全覆盖。人居环境持续改善。文明创建“十大提升行动”*开展，城市管理社会参与机制初步建立，顺利通过国家卫生城市创建暗访评估，接受了全国文明城市创建第二轮测评。一批老旧小区、背街后巷和集贸市场得到整治。中心城区新增绿地面积98.5公顷。在全省率先启动国家生态文明建设示范区创建，成为全国十佳生态文明城市，大气环境质量保持全省前列。泾县、绩溪、旌德纳入国家重点生态功能区。宁国荣获首届中国生态文明奖和第三届全国国土资源节约集约模范县（市）。国家生态县（市）实现市域全覆盖。森林增长工程全面完成。美丽乡村建设和“三线四边”环境治理取得实效，3个县被评为全省美丽乡村建设先进县。12个镇跻身全国重点镇，31个村入围中国传统村落。

（五）着力增进民生福祉，发展成果进一步共享。脱贫攻坚强力推进。实施脱贫攻坚八项行动，*开展“单位包村、干部包户”和“百企帮百村”精准扶贫行动，完成产业项目129个。省定易地扶贫搬迁任务超额完成。全面落实贫困家庭高中学生学杂费减免政策。2.4万余名贫困残疾人得到救助。有望超额完成省下达的45285名贫困人口现行标准下脱贫、12个贫困村出列目标。民生保障水平明显提升。财政民生支出占比达87%。省定民生工程和市政府民生实事全面完成。新增城镇就业4.5万人，登记失业率3.1%。全民参保登记计划全面启动。城乡低保标准稳步提升。保障性住房基本建成率达149.8%，棚改货币化安置率达80%。改造农村危房7460户。社会事业发展“三年行动计划”稳步实施。义务教育学校“全面改薄”年度任务全部完成。新改扩建公办幼儿园14所。城区新建小学3所。中职学生学费减免政策全面落实。宣州荣获全国首批农村职业教育和成人教育示范县。宁国成为全省唯一的国家学前教育改革发展试验区。成功承办世界男排联赛分站赛和全国山地自行车冠军赛。医药卫生体制改革全面深化，医共体试点和家庭医生签约服务等工作扎实开展。宣木瓜、灵芝、黄精入选“十大皖药”。实施全面两孩政策，计生服务管理进一步完善。文化馆一级馆比例跃升50个百分点。村级文化广场示范项目通过文化部验收。宣纸制作技艺荣获“国匠荣耀传承奖”。市残疾人康复中心、市地震台建成，多普勒雷达完成吊装。社会治理得到加强。建成公共信用信息共享服务平台。市级社会服务综合管理平台建成运行，基层网格化管理实现全覆盖。法治宣城、平安宣城建设扎实推进，成为全国“六五”普法先进城市、首批法治城市创建先进单位。在全省率先开通县级网上信访平台，省交办信访积案全部化解。安全生产事故起数和死亡人数继续“双下降”。食品药品安全城市创建扎实推进。G20峰会宣城市安保工作得到国家信访局通报嘉奖。应急管理工作得到加强。人民防空工作跨入全省先进行列。全面完成征兵和退役士兵安置任务，创成全国双拥模范城。海军“宣城舰”命名下水。抗击水阳江中下游严重洪涝灾害取得全面胜利，受灾群众生活得到有效保障；1077户水毁群众住房重建维修任务提前全面完成。

各位代表，2016年是本届政府的收官之年，各项工作全面完成，为本届政府划上了圆满句号。过去六年，我们坚持“11331”发展思路、“好为标准、快字当先”工作基调和“六年并进、六城同创”中心工作，战胜了诸多困难，经受住了严峻挑战和风险考验，宣城大地发生了新变化、增添了新活力，经济社会发展迈上了新台阶。

六年来，我们坚持抓好发展要务，经济实力持续增强。地区生产总值连跨五个百亿台阶，首次突破千亿大关，年均增长10.5%，人均生产总值居全省第8位。财政收入突破200亿元，年均增长15.6%，人均财政收入居全省第5位。累计完成固定资产投资6252亿元，年均增长19.3%。宁国进入全国科学发展百强县，3个县市区跻身中国最具投资潜力中小城市百强。

六年来，我们坚持推进转型发展，质量效益稳健向好。服务业增加值占生产总值比重由36%增至40.4%；战略性新兴产业产值占规模以上工业总产值比重由8.8%升至25.2%，居全省第6位。荣获全国科技进步先进市称号，2家企业获得国家科技进步二等奖。新增省级以上研发平台70个，高新技术产业增加值占规模以上工业增加值比重提高7.1个百分点。单位GDP能耗、GDP建设用地和工业增加值单位耗水量分别下降27.6%、31%、57.5%。

六年来，我们坚持深化改革开放，发展活力不断激发。积极推进国家和省级改革试点，累计完成改革任务268项。市场主体由9.3万个增至16.1万个，民营经济占比达71%，保持全省首位。新增银行机构4家、上市企业5家、“新三板”挂牌企业19家。国内外友好城市达3个。宣城、宁国国家级经济技术开发区转型发展加快。亿元以上项目实际到位省外资金、实际利用外资累计达3584亿元和36.8亿美元，新增对外贸易92.7亿美元。

六年来，我们坚持统筹协调发展，城乡面貌明显改善。城镇化率由43.3%升至52.1%。累计完成城市建设投资798亿元，中心城市建成区面积扩大12平方公里，一批文化、教育、卫生、体育等公共配套设施建成使用。累计完成交通建设投资375亿元，新增高速公路通车里程260公里、一级公路通车里程270公里。合福高铁、水阳江下游近期防洪治理等重大工程建成。美丽乡村建设水平全省领先。“六城同创”成果丰硕，城市美誉度、知名度明显提升。

六年来，我们坚持为民惠民理念，民生水平显著提高。持续加大民生投入，群众获得感明显增强。城镇、农村居民人均可支配收入实现翻番，均居全省第4位。城乡居民养老保险和医疗保险、大病保险实现全覆盖。三甲医院可望实现零的突破。计生工作连续六年被省政府表彰为先进市。公共文化场馆等级率和人均体育场地面积均居全省第3位。连续4年获得全省综治工作（平安建设）先进市称号，信访工作连续6年、安全生产管理工作连续5年获得省表彰，群众安全感满意度连续6年位于全省前列。

六年来，我们坚持转变政府职能，施政能力逐步提升。扎实开展群众路线教育实践活动和“三严三实”专题教育、“两学一做”学习教育、“讲看齐、见行动”和观念更新学习讨论。“放管服”改革持续深化，“三个清单”制度全面实施。市级行政审批事项由496项减至111项。法治政府建设加快，科学决策机制不断完善。自觉接受人大依法监督和政协民主监督，启动政府立法工作，落实协商民主制度；建立市政府负责同志领衔办理制度，议案、建议和提案办理满意率稳步提高。深化政务公开和政务服务，政风建设评价位列全省第二。强化领导干部经济责任审计，加强对公共资金、国有资产、国有资源的审计监督，节支22.4亿元。坚持廉洁从政，减少“三公”经费支出3.1亿元。认真落实“两个责任”，严格执行党风廉政建设制度，持续整治不正之风，严厉查处腐败问题。

各位代表，本届市政府任期的六年，是打基础、利长远、惠民生的六年，也是充满挑战、共克时艰、满载收获的六年。经过六年的发展，宣城已站在新的起点。正如省委书记李锦斌同志在参加省第十次党代会宣城代表团分组审议时指出，宣城经济实力显著提升、发展活力显著增强、基础设施显著改善、绿色生态显著发展、人民生活显著改善，在全省的地位和作用越来越突出。

六年风雨兼程，成绩令人振奋。这是省委、省政府和市委坚强领导的结果，是市人大、市政协监督支持的结果，是历届班子接力奋斗的结果，是全市人民苦干实干的结果。在此，我谨代表市人民政府，向全体人大代表、政协委员，向全市广大工人、农民、知识分子、干部和离退休老同志，向驻宣解放军指战员、武警官兵和政法干警，向各民主党派、工商联、各人民团体和社会各界人士，向关心支持宣城发展的中央和省驻宣单位，向在我市创业奋战的广大投资者、建设者，致以崇高的敬意和衷心的感谢！

回顾六年的发展历程，我们也清醒认识到：发展不足、发展不优、发展不均问题依然突出，尤其是工业支撑力不够，主导产业不大不强，省际交汇区域中心城市的建设还刚开始；自主创新能力不强，制约发展的深层次体制机制弊端尚未根本破除；民生保障水平和社会治理能力亟待提升；少数部门和干部观念更新不够，作风不实、担当不够等问题仍然存在，工作中“互为前提、互为条件”的推诿扯皮现象时有发生，发展环境需要进一步优化，人民群众对我们的工作还有不满意的地方。对此，我们一定要高度重视，采取有力措施，切实加以解决。

二、今后五年的奋斗目标和主要任务

经过六年的发展，宣城已经成功进入全省经济总量“千亿俱乐部”，皖苏浙省际交汇区域中心城市的发展定位更加明确，“双百”中心城市规模为未来发展提供了更加广阔的空间。未来五年，是全市转型突破的关键期，是加快跨越崛起的机遇期，是决胜全面小康的攻坚期。只要我们坚持发展为上、创新为魂、民生为本、脱贫为先、平安为基，只要我们更新观念、担当尽责、锐意进取、真抓实干，就一定能在新的起点上实现新的跨越，一定能确保皖苏浙省际交汇区域中心城市建设取得实效，一定能如期完成市第四次党代会提出的“一个提前、四个提升”宏伟目标。

未来五年政府工作的总体要求是：全面贯彻党的基本路线和十八大以来中央各项决策部署，*贯彻落实习近平总书记系列重要讲话特别是视察安徽重要讲话精神，认真落实省第十次党代会和市第四次党代会确定的各项任务，紧紧围绕“235”工作总体布局，*实施“五大发展行动计划”，聚力打造“四个特色之城”，加快建设皖苏浙省际交汇区域中心城市，全面完成“十三五”规划目标，实现在全省发展方阵中争先进位。

今后五年经济社会发展的主要目标是：

——力争经济总量或人均指标进入全省第一方阵。主要经济指标增幅高于全省平均水平。力争到2021年，生产总值达到1800亿元，按可比价计算年均增长11.5%以上；财政收入达到280亿元，年均增长8％左右；人均生产总值和人均财政收入分别迈上1万美元、1万元台阶；城乡居民收入增长与经济增长保持同步。

——初步建成充满活力的现代产业之城。以战略性新兴产业为先导、先进制造业为主体、现代服务业为支撑、现代农业为基础的优质高效产业新体系初步构建，成为省级创新型试点城市。建成面向长三角地区的新兴制造业基地，工业强市和主导产业地位进一步提升，现代服务业占比超过60%，基本形成一二三产业融合的现代产业发展新格局。

——建成要素汇聚的综合枢纽之城。“芜马宣”城市组群一体发展加快，成为长三角城市群重要中等城市。“双百”中心城市建设迈出坚实步伐，首位度和辐射力进一步提升，中心城区人口达到70万，建成区面积达到80平方公里左右。基本形成以宣城为枢纽、县县通高铁、县县通高速、水陆空协同的立体交通网络。开放合作进一步深化，创新基地和平台建设取得成效，成为科技、人才、资金、信息等要素资源聚集地。

——建成有品有味的文化生态之城。城市文化内涵得到丰富，园林城市特色进一步彰显，全国生态文明先行示范区建设成效明显，“山水诗乡、多彩宣城”品牌魅力显著提升，初步建成生态大公园、文化大观园、旅游大乐园、宜居大家园。创成国家全域旅游示范区、历史文化名城和全省首个国家生态文明建设示范市。

——建成共建共享的平安幸福之城。城乡居民收入持续增长，“人脱贫、村出列”目标全面实现。就业和社会保障体系进一步完善，基本公共服务均等化水平稳步提高，社会文明程度普遍提升，安全生产基础进一步夯实，人民群众获得感持续增强。创成全国文明城市、国家卫生城市、社会治安综合治理优秀城市，成为全省“首安之地”。

今后五年的主要任务是：

（一）实施创新发展行动，培育经济增长新动能。随着宣城经济发展层次的提升，创新发展要求更为迫切，必须把创新摆在发展全局的核心位置，形成以创新为主要引领和支撑的经济发展模式。加速实施全面创新。加快先行先试、积极进取探索、大胆改革创新、优化体制机制，着力解决发展中的矛盾和短板，力争重点领域和关键环节改革取得一批成果、形成一批经验。加快建设创新基地和平台。谋划建设中国宛陵科技创新基地，打造宣郎广宁智创走廊，省民营企业科技创新联盟总部园区初具规模。鼓励企业争设科创平台,争取并支持高校和科研院所来宣设立分校、分院或研发机构，力争省级以上研发平台达150家以上，规模以上工业企业研发机构覆盖率达到40%，研发经费支出占生产总值比重达到2.5%以上，形成一批拥有自主知识产权和核心技术的科技成果及产业化项目，实现“宣城制造”向“宣城智造”转变。积极构建创新型现代产业体系。*实施中国制造2025宣城行动方案，统筹推进“三重一创”建设，高水平建设战略性新兴产业集聚发展基地，力争建成产值超千亿基地1个、超百亿基地10个；实施“百亿企业、千亿产业”工程和“千企升级”计划，培育产值超千亿产业1个、超百亿企业3—5家；高新技术产业、战略性新兴产业、规模以上工业产值均实现翻番。壮大现代服务业，力争文化旅游产业成为主导产业，做大做强文房四宝产业，建成“电商宣城”和15个现代服务业集聚区。旅游经济总量跻身全省前列，宣城文化旅游发展股份有限公司力争上市。构建现代农业产业、生产、经营体系，提升农产品品牌，提高农产品供给质量和效率。培育发展“四新经济”和会展经济、总部经济。实施质量品牌提升工程，建设“质量宣城”。努力打造创新创业人才高地。重点突破京沪宁杭合地区，引进高层次科创团队100个以上。实施宣籍在外优秀人才回归工程，促进创新创业。推进职业院校校企联合办学，加强高技能人才培养和职业技能培训，弘扬“工匠精神”，打造区域技工大市。加强企业家队伍建设，培育一批创新型企业家。

（二）实施协调发展行动，构建统筹联动新格局。统筹城乡、壮大本级、做特县域，形成中心城市与县城功能互补、分工明确、联系密切的有机整体，提升市域发展整体效能。全市常住人口、户籍人口城镇化率分别达到59.2%和37%。提升中心城市能级。坚持产城融合、市区一体，加快宣城和宣州经开区建设、敬亭山旅游度假区、现代服务业产业园区发展，积极推进文教创意产业集聚区、中央商务集聚区、高铁新区建设，完善城市基础设施支撑体系，积极推进区划调整，集聚人口、壮大产业。到2021年，市区地区生产总值、财政收入占全市比重分别达到30%和40%以上。再铸县域经济辉煌。促进县市区经济增速在全省位次稳步提升，着力打造比肩苏浙的经济强县、旅游文化名县、生态示范强县，支持有条件的县市区冲刺全国科学发展百强。支持县域打造宜居宜业、特色鲜明的现代化小城市。着眼全省领先，建成一批全国、省级特色小城镇。整市推进美丽乡村建设。到2019年，实现美丽乡镇建设、中心村建设、农村生活垃圾处理和自然村环境整治“三个全覆盖”，全面建成富有宣城特色的美丽乡村。*推进农村道路畅通工程，修好群众“家门口”的路。加快水利“一规四补”建设，实施灾后水利水毁修复与薄弱环节建设治理三年行动。

（三）实施绿色发展行动，建设生态宜居新家园。绿水青山就是金山银山。要加快国家生态文明先行示范区建设，打造泾旌绩文化旅游生态健康产业走廊，形成生态文明建设和产业发展的宣城样板。加强生态保护和修复。全面落实主体功能区规划，坚持保护优先、自然恢复为主，积极推进绿色生态廊道建设和全国绿化模范城市创建，筑牢生态安全屏障。着力改善环境质量。打好大气、水、土壤污染防治三大战役，实施“洁气、洁水、洁土”工程，加强重点领域污染整治和土壤环境、“三江一湖”流域水环境治理，让宣城更加天蓝、地绿、水净、气爽。加强资源节约集约利用。实行能源和水资源消耗、建设用地等总量和强度双控行动，发展节能环保产业，努力形成绿色生产体系、市场体系、投资体系。强化资源综合利用，大力发展循环经济。积极配合国家和省开展多金属矿、页岩气（油）等矿产资源深度勘查开发工作。推进绿色生活导向工程，创建国家节水型城市、低碳城市。构建市县乡村“四位一体”的环境保护和监管体系，加强生态文明建设绩效评价考核和责任追究等制度建设。

（四）实施开放发展行动，打造内陆开放新高地。积极融入长江经济带，全面学习对接沪苏浙，努力成为推进长三角一体化发展先行区。构建大通道。加快交通网络和枢纽节点建设，建成商合杭铁路宣城段、杭黄铁路绩溪段，完成皖赣铁路芜宣段扩能改造，开工建设宣城至绩溪、宣城至镇江铁路，宣城高铁站和客运总站投入使用，打造区域铁路枢纽。实施公路建设“五大工程”，宣狸、广宁、芜黄高速公路建成通车，建设宣泾高速公路；完成水阳江航道整治和宣州综合码头建设，推进定埠港运营。建成芜宣机场和泾县通用机场，发展民用航空。谋划宣城至芜宣机场、宣城至高淳轨道交通。提升大平台。推进省级以上开发区转型升级。宣城和宁国经开区创成国家级转型升级示范开发区，广德、郎溪经开区争创国家级开发区，宣州经开区创成国家级高新技术产业开发区。实现大通关。深化关检“三个一”合作，建立高效便捷的通关制度，全面实现口岸管理的信息互换、监管互认、执法互助。加强海关监管平台建设，力争建成国家内陆二类开放口岸。培育外贸竞争新优势，力争2021年当年进出口总额达22.5亿美元，实际对外投资累计超过3亿美元。推动大合作。加强与“一带一路”沿线地区合作交流，推进与沪苏浙产业发展融合对接，强化招商选资、招大引强和园区合作共建，加快形成与之产业互补协调发展格局，力争中德合作产业园区、苏皖合作示范园区建设和沪宣“飞地”合作取得成效，引进1—2个百亿级项目。

（五）实施共享发展行动，增进人民生活新福祉。坚持以人民为中心，不断加大民生投入，持续增加居民收入，让发展成果更多更公平地惠及人民。坚决打赢脱贫攻坚战。坚持项目带动、精准施策，扎实推进脱贫攻坚八项行动，确保贫困群众全部脱贫、贫困村全体出列，全力巩固脱贫成果。推动更高质量就业。完善公共就业创业服务体系，大力推进“双创”，新增城镇就业15万人；扎实推进农村“三变”改革，促进农民就地就近就业。完善社会保障体系。实施全民参保计划，基本实现法定人群全覆盖、待遇水平稳步提高，构建社会托底保障体系。基本完成现有的城镇棚户区、城中村和危房改造，加大城镇老旧小区整治和农村危房改造力度，实现城镇常住人口住房保障应保尽保。扩大公共服务供给。*实施社会事业发展“三年行动计划”。实施“健康宣城2030”规划纲要，大力发展健康服务产业，建设健康宣城。大力推进教育现代化，促进各类教育均衡协调发展和质量提升。继续支持合工大宣城校区加快发展。宣城职业技术学院创成地方技能型高水平大学。*推动全民健身，提升全民体质。积极打造国际国内品牌体育赛事。践行社会主义核心价值观。大力发展文化事业，建成省级公共文化服务体系示范区，创成“中国书法城”。优化人口结构，提高人口质量。深化法治宣城、平安宣城建设。完善矛盾纠纷排查调处机制，推进社会服务管理信息化和社会治安防控体系建设。加强社会信用体系建设。*开展“铸安行动”，强化食品药品安全监管，加强综合防灾减灾救灾能力和应急体系建设。

各位代表、同志们，宣城有机遇叠加、区位优越的有利条件，有风清气正、心齐劲足的发展氛围，有勤劳善良、团结务实的干部群众，有不断进取、勇于开拓的创业队伍，这是我们站在新起点、推动新发展、实现新跨越的动力源泉。我们坚信，经过五年的顽强拼搏，一个具有较强实力、充满创新活力、彰显无限魅力的新宣城一定会展现在世人面前。

三、扎实做好2017年各项工作

2017年是实施“十三五”规划的重要一年，是推进供给侧结构性改革的深化之年，是落实“五大发展行动计划”、打造“四个特色之城”的起步之年，也是新一届政府的开局之年。高起点、高标准做好今年各项工作，意义重大、影响深远。我们要坚持稳中求进工作总基调，以提高发展质量和效益为中心，以推进供给侧结构性改革为主线，认真落实“五大发展行动计划”，扎实推进“四个特色之城”建设，努力实现新起点上新发展和新进步。

建议全市经济社会发展的主要预期目标是：地区生产总值增长8.5%左右，财政收入增长保持同步，规模以上工业增加值增长9%左右，服务业增加值增长10%左右，固定资产投资增长11.5%左右，社会消费品零售总额增长11%左右，进出口总额争取实现正增长；城乡居民收入增长与经济增长保持同步；新增城镇就业3.3万人；完成省定节能减排任务。

围绕上述目标，我们将重点做好以下六个方面工作：

（一）坚持创新驱动发展。扎实推动供给侧结构性改革。持续有力推进“去降补”，进一步完善政策措施，推动实体经济稳健向好。更好运用市场化、法治化方式，坚定不移化解过剩产能，加快铜加工、耐磨材料等行业转型升级，妥善处置企业债务，妥善安置企业职工。支持农民进城和个人住房消费，有效盘活闲置资产，促进房地产市场平稳健康发展。扩大棚改货币化安置和公租房货币化保障，完成棚改10000套以上。完善政府性债务风险预警、化解机制，防范化解金融风险。*实施企业上市“翱翔计划”，力争1—2家企业上市报会、5家企业“新三板”挂牌，直接融资80亿元。扩大“4321”政银担、税融通、出口退税贷业务，力争新增贷款130亿元。开展减税清费降本行动，切实减轻企业负担。继续开展“项目提质提效年”活动，推进一批补短板重点项目。实施市重点项目500个以上，完成投资500亿元以上，力争纳入省亿元以上重点项目投资完成率继续保持全省前列。加快±1100千伏古泉换流站等重大基础设施项目和新能源、新材料、电子信息等产业项目建设。提升项目谋划水平，谋划推进重点项目500个以上。增强自主创新能力。完善科技创新政策体系，推动建立省级创新平台10家以上；引进高层次科创团队20个以上，争取省扶持团队2—3个。启动建设中国宛陵科技创新基地，开工建设省民营企业科技创新联盟总部园区，加快宣城智谷软件产业园建设，力争国家机械科学研究总院宣城分支机构、安工大宣城工业技术研究院、美国加州大学戴维斯分校国际技术转移（宣城）中心等挂牌。实施产学研合作项目200个左右。新增授权发明专利600件以上。集聚发展新兴产业。加快战略性新兴产业发展，突出抓好宁国省级基地和5个市级基地建设，力争战略性新兴产业产值增长11%左右。实施高新技术企业培育发展专项行动，新认定高新技术企业35家以上，高新技术产业增加值增长15%以上。做大做强主导产业。力争三大工业主导产业产值突破千亿。继续推进“千企升级”，工业技改投资增长10%以上。力争产值超亿元企业达400家、省级专精特新企业达100家，新增“小升规”企业100家。推进两化融合和质量品牌提升。实施智能制造“315”工程，提升企业智能化技术水平。加强质量品牌和服务标准化建设，新增省级以上两化融合管理体系贯标试点企业6户；新申报中国驰名商标2件，新增省著名商标28件、安徽名牌产品12件以上。积极推进文房四宝产品质量监督检验中心建设。支持宁国、广德分别创建全国、全省质量强市示范市（县）。扩大内外销产品“同线同标同质”从食品向消费品扩展。加速发展现代服务业。继续开展“文化旅游提升年”活动，推进国家全域旅游示范区创建，文化产业增加值、旅游总收入分别增长8%和18%以上。宣城文化旅游发展股份有限公司正常运营。推进敬亭山、大桃花潭、太极洞、青龙湾、观天下等5A景区创建，新创1—2家4**景区。加大资源整合力度，着力打造一批精品线路。加快中华诗文化博览园和红楼梦文化大观园、中国园子、国际慢城等项目建设。精心举办第八届“文房四宝文化旅游节”。实施“电商宣城”行动计划，加快快递物流业发展，加强农商互联对接。大力发展金融产业，做好与民生银行、正海资本、国厚资产管理等对接，力争新引进1家银行和2家本地注册的基金；推进普惠金融发展，力争村镇银行县域全覆盖。发展股权融资和投资。加快发展文化创意、信息技术、仓储物流、居家养老、健康养生、教育培训等服务业。培育商贸流通企业，新增限额以上商贸流通企业15家以上。

（二）大力推进城乡统筹。加快中心城市产城融合发展。认真做好城市总体规划编制国家和省级创新试点，按照中心城市“双百”发展定位，完成新一轮城市总体规划修编。全面推进“多规合一”工作，完成城市空间规划编制。加快宣城经开区转型发展，新签项目55个，新开工工业项目22个、投产20个，培育新能源新材料百亿产业集群，确保主要经济指标增幅保持全市前列；加快宣州“两区四园”转型升级，支持宣州经开区争创国家级高新区。推动宣城现代服务业产业园区发展，加快新塘羽绒产业园、建材（钢材）物流交易中心、电子商务与快递业协同发展集中区等项目建设。开展“城市建设突破年”活动。在主干路网建设、棚户区改造、服务功能完善、征地拆迁、品质提升、防洪保安上求突破，实施中心城区建设项目223个，完成投资150亿元。续建、新（改）建水阳江大道闭合段、阳德路、宛陵东路等76条道路，力争用两年时间闭合城区“一环、二环”，实现水阳江大道全线贯通。加快市全民健身中心、游泳馆等项目建设和宛溪河综合整治二期及北门棚户区改造。加快市科技馆项目前期工作。打造以叠嶂路中心商务区等为重点的城市精品街区。加快海绵城市、地下综合管廊建设和地下空间综合开发利用。确保完成房屋征收4000户、60万平方米以上，强化违法建设管控。*实施城区老旧小区、背街后巷、“三车”运输秩序等专项整治，加强城市立面改造和市容管理提升，继续争创全国文明城市，创成国家卫生城市。加快新型智慧城市建设，扩大智慧政务、教育、医疗、旅游、工业互联网+等应用。强力推进双桥联圩、城东联圩、敬亭圩防洪治理工程及城区排涝泵站建设，提升城乡防洪排涝整体标准。推动县域经济振兴和特色小城镇建设。安排2亿元专项资金，支持县市区对照沪苏浙赶超目标，发展各具特色的板块经济。支持广德经开区升格。*实施城镇“三治三增三提升”行动。加快推进17个市级特色小镇培育创建，重点推进宣纸、宣笔、宣砚、徽墨、中德智造等特色小镇建设。推进现代农业发展。争创省级示范联合体8个、示范合作社7个、示范家庭农场20个以上。加强宣城国家农业科技园区等农业产业化示范区建设，发展农产品精深加工，力争加工产值达800亿元。稳定粮食生产，推进“放心粮油”“主食厨房”工程。加快宣木瓜、灵芝、黄精等中药材产业基地建设和茶叶、蔬菜等产业发展。加快宣州现代功能农业科创园建设，组建蓝莓科研中心和长三角植物功能食品研究中心。实施“生态宣城·绿色产品”品牌工程，新认证“三品一标”农产品60个，争创国家级、省级农产品质量安全县和省级以上农业标准化示范区。认真做好政策性农业保险工作，扩大特色农业保险覆盖面。加快美丽乡村建设。强化涉农资金整合，完成31个乡镇政府驻地建成区整治，建设省级中心村55个、自主建设中心村60个。继续开展村级环境整治，健全“三线四边”环境治理长效机制。改善农村基础设施条件。加快水利水毁修复与薄弱环节建设治理，力争开工牛岭水库、港口湾水库灌区等项目，扎实推进水阳江中游治理、凤凰山水库等项目前期工作。继续实施青弋江等中小河流治理、扬溪源等中小水库、青弋江灌区三板桥闸和排涝泵站除险加固达标工程。加快电力基础设施建设，续建新建110千伏以上电网项目12个，完成农网改造升级任务。加强镇村生活垃圾、污水收集处理设施建设，规范运营管理。

（三）推进绿色低碳发展。强化生态建设保护。继续推进水阳江生态蓄水工程建设，实施中央城市生态公园、城市水环境治理等项目，力争开工青弋江引水入城活水工程。开展稳绿增效和市区园林绿化提升行动，推进“绿色矿山”建设。建立重点区域、流域跨界环境污染和生态破坏联合防治协调机制。全面推进生态文明建设示范市创建，力争达到国家考核标准。大力实施“三洁”工程。推行清洁能源技术，强化烟尘、扬尘整治，完成黄标车淘汰任务。实施工业企业全面达标排放计划，强化污染源监管。全面落实“河长制”，加强水污染防治，强化集中式饮用水水源地保护。开展土壤环境质量监测与评估，强化农业面源污染、重金属污染防治，严格控制无序种植养殖，实施化肥农药零增长行动。积极推进绿色发展。开展绿色制造体系建设试点，加快传统工业绿色化改造，推行清洁生产，大力发展环保产业和循环经济。实施节能预警调控、重点用能单位能源消费监管制度，提升企业能源管理水平。完成市县乡三级土地利用总体规划修改完善和全市矿产资源总体规划编制工作，强化闲置低效土地处置。加强秸秆禁烧工作，推进农作物秸秆综合利用。推广绿色生活方式。

（四）深化重点领域改革。抓好重点改革突破。强化要素资源保障、政策落实，促进民间投资，推动民营经济发展。深化“营改增”、国税地税征管体制、公共资源交易监管体制和商事制度改革。深化公立医院改革和医保支付方式改革、资源价格和公共事业价格改革。实施城管执法体制改革。稳妥实施事业单位改革和机关事业单位工作人员养老保险制度改革。创新政府科技管理体制，探索知识产权综合行政执法。稳步推进农村集体产权制度改革，巩固农村土地“三权”分置改革成果，发展多种形式适度规模经营，扩大“三变”改革试点规模。*推进农房抵押贷款试点。深化小型水利工程管理体制改革。全面完成国有林场改革，加强国家集体林业综合改革试验示范区建设，组建林权收储中心。积极稳妥推进供销合作社综合改革。优化完善体制机制。建立统一高效、指挥有力的重大项目谋划、推进、建设和招商引资工作指挥调度机制。组建并实体化运作城市大建设指挥调度体系，完善城市建设管理体制机制，深化城市治理体制改革。增强市国投公司“造血”功能，推进转型发展、做大做强。推进宣城现代服务业产业园区市场化运作、公司化运营，探索园区综合执法和行政服务新路径。进一步理顺市与宣州区、宣城经开区财税体制，实现权责利相统一，促进市区一体发展。

（五）提升开放合作水平。开展“交通建设提升年”活动。完成交通建设投资60亿元以上。建成宣狸高速，加快建设广宁高速，开工建设芜黄高速宣城段，谋划宣泾高速、申嘉湖高速宁国段建设。建成S214定十路和S215绩溪至歙县段2条一级公路，加快建设G318宣十路等13条国省干线公路，力争开工建设S104宣港路等5条国省干线公路。完成国省道路面改善和生命安全防护工程200公里、农村道路畅通工程1000公里。加快推进水阳江航道整治和宣州综合码头建设工程。建成宣城客运总站。加快皖赣铁路扩能改造芜宣段、商合杭铁路宣城段、杭黄铁路绩溪段建设和芜湖至广德铁路电气化改造，启动巷口桥铁路物流中心建设，加快推进宣城至绩溪铁路、宣镇铁路和武杭高铁宣城段前期工作。推进芜宣机场建设。加快合作发展。积极参与长江经济带区域合作，*落实与沪苏浙城市合作协议。推动中德合作产业园区建设，推进白茅岭、军天湖沪宣“飞地”合作，积极打造苏皖合作示范园区。加强与省地矿局、海螺集团的战略合作，共建地矿产业园等项目。开展“招商引资实效年”活动。抢抓杭州承办亚运会契机，承接产业整体转移。修订完善政策，推进精准化、专业化和市场化招商，强化调度、督查、考核、奖励，提升招商引资实效。力争新引进10亿元以上项目10个以上，确保开工、竣工、投产项目个数和实际形成固定资产投资额均增长10%以上，实际利用外资增长7%。努力扩大对外贸易。继续实施外贸“三百工程”，抓好加工贸易和出口结构优化，积极培育外贸企业集聚区。推动卫浴产品转型升级。支持郎溪创建出口食品农产品质量安全示范区。加快外贸综合服务和跨境电商平台建设。确保皖东南保税物流中心和宁国公用型保税仓库建成运营。推动宣城无水港实体化运作，积极申报国家二类口岸。支持中鼎集团等企业开展全球并购、中国整合、宣城制造。

（六）着力补齐民生短板。全力实施脱贫攻坚。坚持以市场化为导向，以产业项目为抓手，完善精准帮扶举措，*推进脱贫攻坚八项行动，确保97个贫困村出列。加强就业和社会保障。扎实推进“双创”工作，力争新登记民营企业3600户、个体工商户1.4万户以上。实施“创业江淮”、高校毕业生就业促进、农民工职业技能提升等计划，健全就业援助制度，促进各类群体就业。巩固城镇居民大病保险全覆盖成果，推进工伤保险扩面，提高城乡低保标准和特困供养人员供养标准。完善社会救助和保障标准与物价上涨挂钩联动机制。大力发展社会事业。实施第三期学前教育行动计划。加快市区中小学建设，开工建设市十六小，重建市五中，完成市二中、三中、信息工程学校改扩建工程。推进宣城中学打造“皖南名校”工程。全面改善农村薄弱学校办学条件。引进优质教育资源，嫁接名校品牌，加快普通高中多样化、特色化发展。推进合工大宣城校区图书馆、实验楼等项目建设。加快职教园区和市国家综合档案馆·宣城职院科技创新中心建设。积极推进教师“无校籍”管理，促进师资均衡配置。促进民办教育健康发展。加快推进市、县体育基础设施“五个一”工程建设。举办市第二届全民健身运动会。精心承办亚洲山地自行车锦标赛。健全医疗服务体系，推进医共体建设。加快市人民医院外科急诊大楼和市第四人民医院、市妇幼保健院建设，开工建设市公共卫生检验检测中心。积极引进沪苏浙和省内大医院设立分院。增强基层公共卫生服务能力。推动人口计生工作转型发展。继续加强公共文化服务体系建设，推进乡镇（社区）综合文化服务中心和农民文化乐园建设,扩大农家书屋数字化覆盖面。加强古建筑古村落保护和利用。市博物馆新馆和市规划馆对外开放。举办第二届新创作皖南花鼓戏及小品小戏调演。完善市残疾人康复中心服务功能。高质量做好第三次全国农业普查工作。健全留守儿童、妇女、老年人和残疾人关爱服务体系，加强未成年人保护。发展社会福利和公益慈善事业，做好民族宗教、对台事务、外事侨务、地方志、气象等工作。提升社会治理水平。认真开展“七五”普法，提高公共法律服务水平。强化社区基层组织建设。加强法律援助和社区矫正工作。启动市级调解中心建设。*推进“一村一警”包村联系工作，推进综治维稳信访工作中心（站）规范化，完成市级综治视联网建设，试点实施“雪亮工程”，加强网络安全管理，严密防范和依法打击各类违法犯罪活动。着力源头防范、畅通渠道、落实责任、依法处置、案结事了，打造阳光信访、法治信访、责任信访。*推进“铸安行动”，建立安全生产责任保险制度，强化安全隐患排查治理，健全应急救援体系，有效遏制安全生产事故。扎实开展食品药品安全风险分级管理和“四品一械”专项整治，创建食品药品安全城市、食品安全示范县。健全城市应急联动指挥体系和监测预警应急机制，加强防灾减灾救灾和应急管理工作。支持人民团体参与社会管理和公共服务。支持驻宣部队改革和建设，加强国家安全教育、全民国防教育和国防动员、人民防空工作，推进军民融合深度发展。继续做好双拥共建和优抚安置工作，巩固发展军政军民团结。

在抓好省定民生工程基础上，市政府还将办好10项民生实事：①市人民医院创成三甲医院；②启动“城市源水”工程；③完成市区3处停车场建设；④实施部分老旧小区改造提升和部分道路及背街后巷杆线整治；⑤对城区“三无”对象中的特困供养人员发放居家养老护理补贴；⑥对100名贫困精神类残疾人提供住院补贴；⑦实施新生儿疾病筛查；⑧实施5个城区保障性蔬菜基地能级提升工程；⑨在市区建设20个蔬菜直销点；⑩实施市区集中供热工程。

四、全面提高政府治理能力和水平

站在新的历史起点，使命召唤我们励志前行，重任要求我们勇于担当。*落实“五大发展行动计划”、聚力打造“四个特色之城”，实现宣城发展再出发，政府必须创新思维，提升治理能力和治理水平，实现政府工作有新作为、宣城发展有新气象。

（一）坚持履职尽责的法治思维和法治方式。坚持“法无授权不可为、法定职责必须为”，深化法治政府建设，着力构建办事依法、遇事找法、解决问题用法、化解矛盾靠法的法治良序。健全完善依法、科学、民主决策机制，实行重大决策终身追究制、责任倒查制，提高决策科学化、民主化和法制化水平。继续推进政府规章制定工作。坚持重大事项及时向市委请示报告，主动接受人大法律监督、工作监督和政协民主监督，认真听取人大代表、政协委员和民主党派、工商联、无党派人士、人民团体的意见建议，严格执行人大及其常委会决定决议，提高人代会议案和人大代表建议、政协委员提案办理质量。深化行政执法体制改革，推进重点领域综合执法，规范行政执法行为。发挥行政复议监督纠错功能，健全行政机关负责人出庭应诉情况通报制度。

（二）争当创新实干的行动英雄。巩固“讲看齐、见行动”和观念更新学习讨论成果，始终保持奋发有为、争先进位的精神状态，善于捕捉和把握发展机遇，努力在新一轮改革发展大潮中勇立涛头。天上不会掉馅饼，政府系统全体人员要撸起袖子加油干，坚持“我的阵地我坚守”，积极主动谋发展，勇于担责干工作，想方设法干成事，不达目的不罢休，真正成为实干兴宣的行动英雄。强化政府执行能力，践行一线工作法，实干苦干、务实重行，落实“三查三单”“四督四保”和精准帮扶重点企业、重点项目制度，每季度开展集中开工、竣工、投产活动，推动重点任务、重大工程落细落实落地。突出想干事、敢干事两个关键点，坚持以解决矛盾问题为导向，建立健全正向激励、容错纠错机制，建设市政府重点工作调度监测信息化系统，提升行政效能。加强政务督察、行政监察和问责问效，下大气力治理“浮庸懒慢”顽疾，严厉惩处不作为、不担当行为。

（三）打造高效优质的服务环境。深化“放管服”改革，开展行政审批制度改革“回头看”，大力推行并联审批。深化清单制度建设，打造“三个清单”升级版；建立公共服务和行政权力中介服务清单制度。积极推进“互联网＋政务服务”，发挥市政府权力清单运行平台、市社会服务管理信息化平台作用，全面推进政务服务标准化、便捷化，最大程度利企便民。全面推进政务“五公开”，强化政策解读和政策落实，加强电子政务和“一网双微一通”建设，继续办好“政民互动”“在线访谈”等栏目，加强“政府服务热线”工作，及时回应社会关切。完善政府守信践诺机制，建立政府失信责任追究和赔偿、投诉、救济等制度，以政务诚信促进商务诚信和社会诚信。积极构建“亲”“清”新型政商关系，依法有效保护各种所有制经济组织和公民财产权，维护企业合法权益，保护企业家精神，增强企业家信心。

（四）建设人民满意的廉洁政府。坚持不忘初心，增强“四个意识”特别是核心意识、看齐意识。落实全面从严治党要求，强化党风廉政建设“两个责任”。坚决把纪律和规矩挺在前面，严格遵守党章党规党纪。自觉践行“三严三实”，持续改进工作作风，弛而不息正风肃纪。完善全口径预算管理和预决算公开长效机制，加强财政资金监管，进一步降低行政成本，确保“三公”经费只减不增。加大力度精简文山会海，把更多的精力和时间用在抓发展上。健全惩治和预防腐败体系，进一步加强对行政权力和重点领域、重点工程、关键环节的监督管理，推进领导干部履行经济责任审计监督全覆盖，坚决整治、查处侵害群众利益的不正之风和腐败问题，做到干部清正、政府清廉、政治清明。

各位代表，同志们！新征程蕴含新希望，新蓝图昭示新辉煌。舟至中流，更需击楫勇进、逐浪前行。让我们更加紧密团结在以习近平同志为核心的党中央周围，在中共宣城市委的坚强领导下，勠力同心、开拓创新，干在实处、走在前列，为加快建设皖苏浙省际交汇区域中心城市、如期实现“一个提前、四个提升”宏伟目标、在全省发展方阵中争先进位而努力奋斗，以优异成绩迎接党的十九大胜利召开!
